# Synthesis and Characterization of ZnO-SiO_2_ Composite Using Oil Palm Empty Fruit Bunch as a Potential Silica Source

**DOI:** 10.3390/molecules26041061

**Published:** 2021-02-18

**Authors:** Fida’i Rahmat, Yap Wing Fen, Muhammad Fahmi Anuar, Nur Alia Sheh Omar, Mohd Hafiz Mohd Zaid, Khamirul Amin Matori, Rahayu Emilia Mohamed Khaidir

**Affiliations:** 1 Institute of Advanced Technology, Universiti Putra Malaysia, UPM Serdang, Selangor 43400, Malaysia; fidai.rahmat95@gmail.com (F.R.); nuralia.upm@gmail.com (N.A.S.O.); rahayuemilia.upm@gmail.com (R.E.M.K.); 2 Department of Physics, Faculty of Science, Universiti Putra Malaysia, UPM Serdang, Selangor 43400, Malaysia; fahmianuar6323@gmail.com (M.F.A.); mhmzaid@upm.edu.my (M.H.M.Z.); khamirul@upm.edu.my (K.A.M.)

**Keywords:** oil palm empty fruit bunch, ZnO-SiO_2_ nanocomposite, silica, structural, optical

## Abstract

In this paper, the structural and optical properties of ZnO-SiO_2_-based ceramics fabricated from oil palm empty fruit bunch (OPEFB) were investigated. The OPEFB waste was burned at 600, 700 and 800 °C to form palm ash and was then treated with sulfuric acid to extract silica from the ash. X-ray fluorescence (XRF) and X-ray diffraction (XRD) analyses confirmed the existence of SiO_2_ in the sample. Field emission scanning electron microscopy (FESEM) showed that the particles displayed an irregular shape and became finer after leaching. Then, the solid-state method was used to produce the ZnO-SiO_2_ composite and the samples were sintered at 600, 800, 1000, 1200 and 1400 °C. The XRD peaks of the Zn_2_SiO_4_ showed high intensity, which indicated high crystallinity of the composite. FESEM images proved that the grain boundaries were larger as the temperature increased. Upon obtaining the absorbance spectrum from ultraviolet–visible (UV–Vis) spectroscopy, the energy band gaps obtained were 3.192, 3.202 and 3.214 eV at room temperature, 600 and 800 °C, respectively, and decreased to 3.127, 2.854 and 2.609 eV at 1000, 1200 and 1400 °C, respectively. OPEFB shows high potential as a silica source in producing promising optical materials.

## 1. Introduction

In recent years, environmental issues have been becoming increasingly more important in Malaysia and all over the world. The palm oil industry is aware of the environmental pollution being caused and is striving towards quality and environmental conservation through sustainable development and cleaner technology approaches. The largest waste materials from palm oil mills, namely oil palm empty fruit bunches (OPEFBs), are extremely abundant, renewable and readily available lignocellulosic materials. Therefore, there is a need for sustainable waste management and to convert the waste into useful materials.

Silica (SiO_2_) is a material with high porosity and a large surface area. It is widely used in fillers, catalysts and pharmaceutical materials [1,2,3]. In industry, SiO_2_ can be produced by using sodium silicate, where the production of sodium silicate requires a large amount of energy to smelt quartz sand and sodium carbonate [4]. Consequently, efforts and ideas have been developed to look for alternatives. Extensive studies have attempted to extract silica from various agriculture waste products such as rice husk, corn cob ash and coconut and palm waste [5,6,7,8,9]. To the best of our knowledge, a study on OPEFBs as a silica source has yet to be explored.

There are a wide range of industrial applications where silica is associated with metals and where the family of silicates is formed. For instance, zeolite clinoptilolite or alumino-silicate (network of AlO_4_ and SiO_4_) have excellent detoxifying and anti-inflammatory properties that can be used in many industrial applications ranging from environmental remediation to oral applications [10,11]. Another silica-metal-based material, ZnO-SiO_2_, has been receiving growing attention from researchers owing to its interesting application in glass ceramics, technical glasses and optical glasses [12,13]. It has excellent luminescence properties in blue, green and red spectral regions [14]. On the other hand, when heat treatment is applied, zinc silicate can be formed [15,16,17]. Zinc silicate has emerged as a good host material in plasma display panels, cathode ray tubes, laser crystal and electroluminescent devices [18,19,20,21].

Due to the large amount of energy needed to produce silica from quartz and sodium carbonate, researchers are aiming to develop alternatives to fabricate ZnO-SiO_2_ by using environmentally friendly silica sources [22,23,24]. OPEFB is envisaged as an alternative for the extraction of silica to produce novel ZnO-SiO_2_ materials. This move will definitely have a positive impact on our ecological system by reducing massive waste from palm oil mill productions every year. In this study, the potential of OPEFB waste as a silica source is investigated, and the structural and optical properties of the ZnO-SiO_2_ composite derived from OPEFB waste are reported.

## 2. Results

### 2.1. Palm Waste Silica

#### 2.1.1. XRF Analysis

The OPEFB was burned at three different temperatures, that is, 600, 700 and 800 °C, to produce palm ash samples. The chemical composition of palm ash samples was analyzed using energy dispersive X-ray fluorescence (EDXRF). The chemical composition of the samples is shown in Table 1.

The OPEFB that was burned at 800 °C showed the highest percentage of SiO_2_. Therefore, it was selected for further processing to eliminate all impurities and increase the percentage of SiO_2_ element in the sample using acid treatment. Table 2 shows the comparison of the sample’s chemical composition before and after leaching.

These results indicate that leaching can increase the percentage of SiO_2_ in palm ash and does not fully eliminate other metal impurities in the sample. This is because sulfuric acid leaching can dissolve the metal elements in the sample. The mechanism involves the existence of carboxyl groups in sulfuric acid that tend to donate protons, hence forming the negatively charged carboxyl group. These negatively charged carboxyl groups can form stable complexes with positive charged ions, Ca^+^ and K^+^, that are present in the palm ash [25]. This is the reason why the impurities of the palm ash were reduced, as can be observed in Table 2.

#### 2.1.2. XRD Analysis

**Figure 1:**
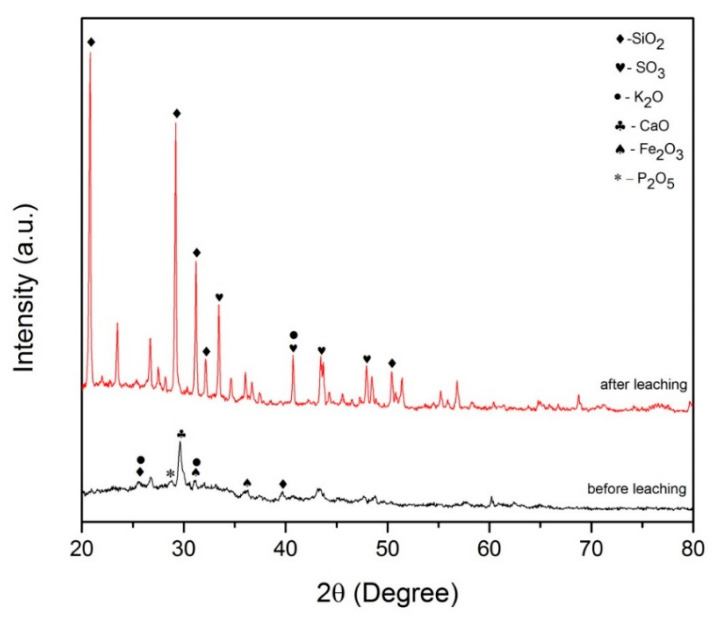
X-ray diffraction (XRD) results for palm ash before and after leaching.

The results of the X-ray diffraction pattern of the palm ash waste before and after leaching are shown in Figure 1. From the graph, it can be seen that there was one obvious strong peak before the leaching process, i.e., CaO at 29.76°. Next, the SiO_2_ element can be found at 26.74° and 39.38° with a lower peak intensity, which has a hexagonal crystal structure. Other elements that can be found with lower peak intensities are K_2_O, P_2_O_5_ and Fe_2_O_3_ with peaks at 25.78°, 29.55° and 40.32°, respectively. These XRD results can be proven by the XRF analysis results shown before.

The X-ray diffraction results after leaching show that more peaks appeared with a higher intensity. The major peaks belonged to SiO_2_ which has a rhombohedral crystal structure at 20.93°, 29.18°, 31.10° and 32.87°. At 40.61°, K_2_O appeared with a hexagonal crystal structure. Another element detected was SO_3_ which has an orthorhombic crystal structure at peaks 32.34°, 40.36°, 43.83° and 47.99°. The presence of SO_3_ was due to the reaction of sulfuric acid used for leaching. These results indicate that leaching can increase the percentage of SiO_2_ in palm ash and remove part of the metal impurities in the sample [25].

#### 2.1.3. FESEM Analysis

**Figure 2:**
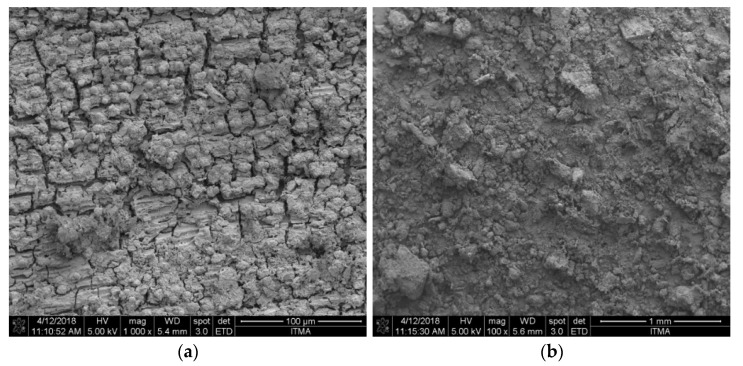
Field emission scanning electron microscopy (FESEM) images of palm waste ash burned at 800 °C (**a**) before and (**b**) after leaching.

The surface morphology of the palm waste silica before and after the leaching process with sulfuric acid is presented in Figure 2. As shown in the figure, the results show that both of them have irregular medium-sized particles distributed with a crushed shape structure. Figure 2b shows particles in the sample that are finer compared to Figure 2a. This is because the leaching process eliminated most of the metal composition in the sample, thus providing finer surface of particles.

### 2.2. ZnO-SiO_2_

#### 2.2.1. XRD Analysis

**Figure 3:**
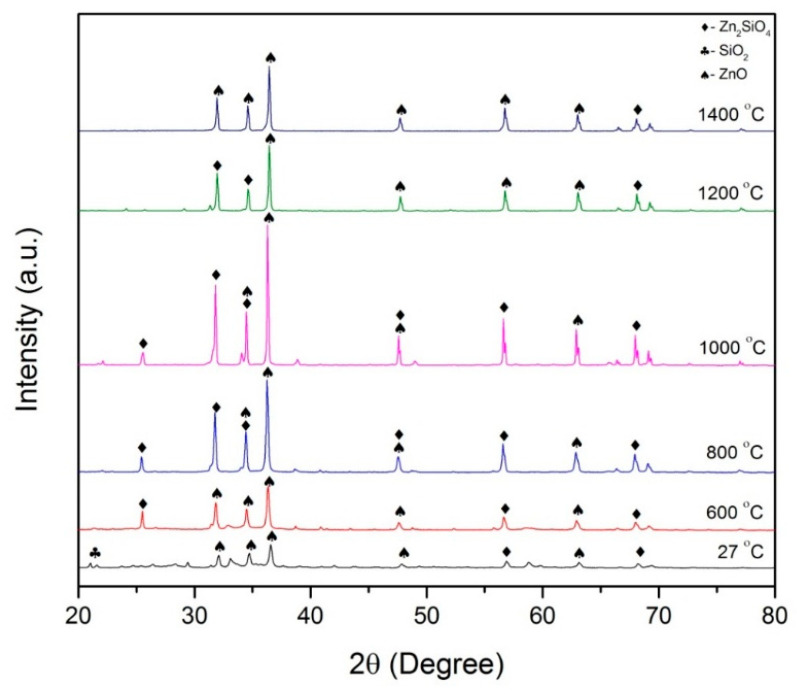
XRD analysis of ZnO-SiO_2_ for different sintering temperatures.

The results of the X-ray diffraction pattern of ZnO-SiO_2_ are shown in the Figure 3. From the graph, it can be seen that the elements SiO_2_ and ZnO appeared in the sample before it was sintered. At 21.09°, a very small peak appeared, indicating the presence of SiO_2_ with an orthorhombic crystal structure. Another element presented in the sample at room temperature is ZnO, which was positioned at 31.85°, 34.55° and, for the highest peak, 36.36°.

At 600 °C, the element of ZnO started to appear at 31.75° followed by 34.44°, and the highest peak of ZnO is at 36.25°. ZnO is hexagonal in structure. The diffraction peaks of single, well-crystallized ZnO phase are clearly obtained (found in excess compared to the stoichiometric amount required for the formation of Zn_2_SiO_4_, where 2ZnO + SiO_2_ = Zn_2_SiO_4_). At a temperature of 800 °C, the intensity of ZnO at peaks 31.82°, 34.47° and 36.19° is higher compared to at the temperature of 600 °C. As the temperature increases, the ZnO nanocrystals may grow to larger sizes but the process is hindered by the surrounding SiO_2_ matrix [17,26]. This causes the SiO_2_ element peak to start to disappear from the graph. As the temperature increased to 1000 °C, strong and high-intensity peaks appeared at 31.77°, 34.42° and 36.25°. The formation of Zn_2_SiO_4_ has the highest peak at this temperature compared to 600 and 800 °C. It is believed that ZnO is the dominant diffusing species in ZnO/SiO_2_ at temperatures greater than 700 °C. For the temperatures 1200 and 1400 °C, the intensity of peaks at 31.85°, 34.55° and 36.36° started to decrease after a temperature of 1000 °C. The decrease in the peak intensity can be attributed to the collapse of the Zn_2_SiO_4_ tetragonal structure. This is because the formation of Zn_2_SiO_4_ started at room temperature until 800 °C, and this increases the crystallinity of the samples, thus increasing the intensity of peaks [19]. The collapse of Zn_2_SiO_4_ formation with the remaining ZnO and SiO_2_ phase at higher temperature causes the crystallinity to decrease and reduces the intensity of peaks at temperatures of 1000 to 1400 °C.

#### 2.2.2. FESEM Analysis

**Figure 4:**
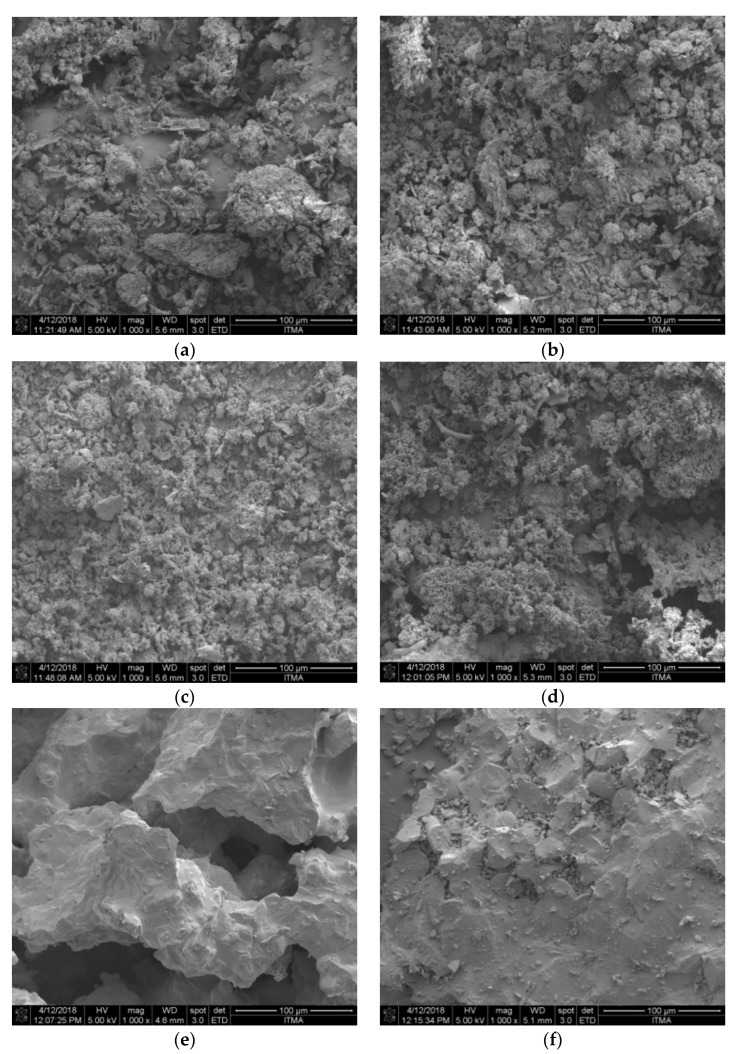
FESEM images of ZnO-SiO_2_ sintered at (**a**) 27, (**b**) 600, (**c**) 800, (**d**) 1000, (**e**) 1200 and (**f**) 1400 °C.

The surface morphologies of ZnO-SiO_2_ that was sintered at various temperatures are shown in Figure 4. All of the SEM images are shown at 1000× magnification. It was observed that the prepared sample at room temperature consisted of particles with numerous little fragments on the surface, not uniform, and has irregular particle size distribution. Next, the microstructure shows that the crystal grains are still in irregular shape at 600 °C and started to aggregate among each other after being heated up to 800 and 1000 °C. When the sintering temperature increased to 1000 °C, the particles start to look like melted forms. However, the microstructure revealed that there is little crystal growth on the sample surface, which indicates the crystals’ formation at the sintering temperatures of 1200 and 1400 °C. At high temperature, ZnO-SiO_2_ becomes granular and has a homogenous distribution, where hexagonal and rectangular shapes can be observed. Overall, the shape of the grains structure is irregular and distributed homogenously for all samples. It can be observed that there is an increment in the grain size with the increase in sintering temperature, as seen from the crystal growth pattern [27,28].

#### 2.2.3. Optical Studies

**Figure 5:**
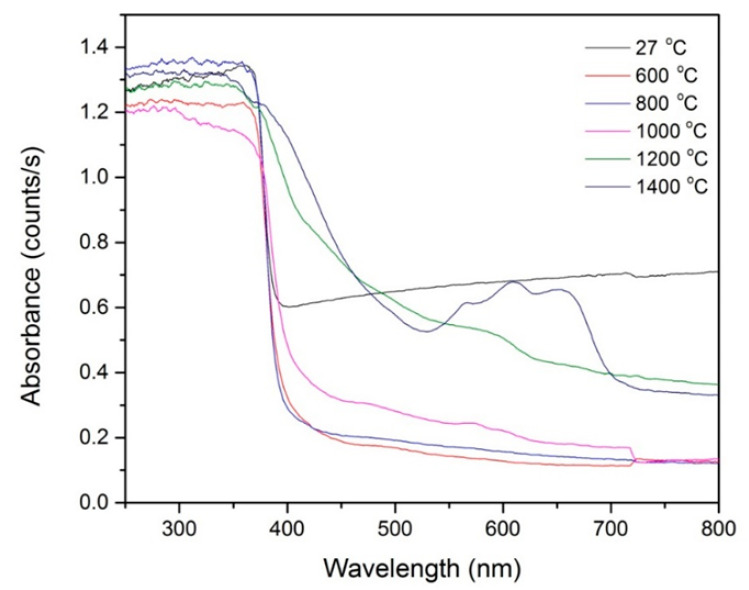
Absorption spectra of ZnO-SiO_2_ at 27, 600, 800, 1000, 1200 and 1400 °C.

The optical properties of ZnO-SiO_2_ at different sintering temperatures were measured using a UV–Visible spectrometer in the range of 200 to 800 nm as shown in Figure 5. It is shown that intensive absorption occurs in the range of 250 to 370 nm for all temperatures. However, when the sample was sintered at 1400 °C, the absorption edge of the sample shifted towards the longer wavelength due to the crystallization of the sample enhancing the absorption of redshift.

Next, the absorbance coefficient was calculated to determine the energy band gap. The absorbance coefficient is given as:

(1) α=2.303A

where the unit of *α* is m^−1^. The energy band gap of these composites can be calculated from the Tauc relation [29,30]:

(2) $$\alpha =\frac{k{\left(hv-E_{g}\right)}^{n}}{hv}$$

where *E_g_* is the energy band gap, *k* is proportional constant, *h* is Planck’s constant, *v* is the frequency of the incident photon and *hv* is the photon energy. The exponent *n* represents the type of transition, where *n* = ½ for direct transition while *n* = 2 for indirect transition. In order to obtain the both direct and indirect energy band gaps, Equation (3) was rearranged to yield:

(3) $${\left(\alpha hv\right)}^{\frac{1}{n}}=k(hv-E_{g})$$

**Figure 6:**
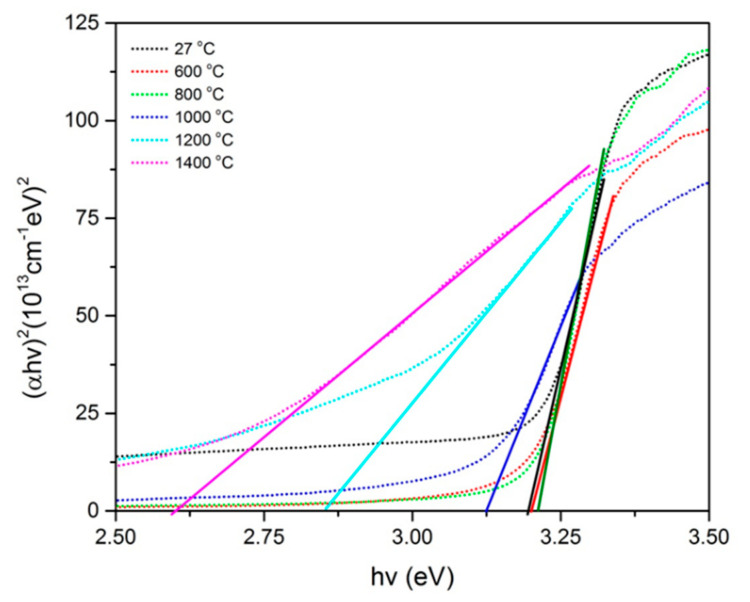
Plot of (*αhv*)^2^ against *hv* of Zn-SiO_2_ composite sintered at various temperatures.

**Figure 7:**
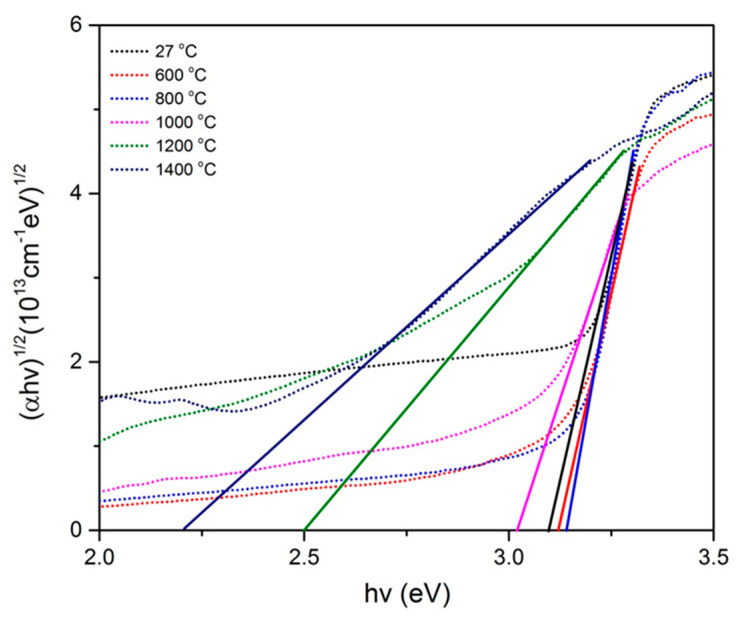
Plot of (*αhv*)^1/2^ against *hv* of Zn-SiO_2_ composite sintered at various temperatures.

By substituting *n* = ½ and *n* = 2, both graphs of (α*hv*)^2^ versus *hv* and (α*hv*)^1/2^ versus *hv* for all sintering temperatures of the samples were plotted, as shown in Figure 6 and Figure 7, respectively. From the graphs, each best fit line was drawn until it reached the *x*-axis, where the intersection gives the value of the direct band gap (Figure 6) and the indirect energy band gap (Figure 7). From Figure 6, the energy band gap of unsintered ZnO-SiO_2_ was found to be 3.192 eV. At 600 and 800 °C, the energy band gaps of the sample were 3.202 and 3.214 eV, respectively. The energy band gap started to drop at temperatures of 1000, 1200 and 1400 °C to 3.127, 2.854 and 2.609 eV, respectively. Meanwhile, for Figure 7, the energy band gap of unsintered ZnO-SiO_2_ was 3.097 eV. At 600 and 800 °C, the energy band gaps of the sample were 3.120 and 3.141 eV, respectively. The energy band gap started to drop at temperatures of 1000, 1200 and 1400 °C to 3.027, 2.500 and 2.204 eV, respectively. The band gap energy increased from room temperature until 800 °C as the crystallinity of the samples improved. This is in agreement with previous studies which revealed that the enhancement of the samples’ crystallinities can cause the reduction in delocalized states, which led to lesser defects, thereby promoting an increase in the band gap values [31]. It started to decrease from 1000 to 1400 °C because the formation of Zn_2_SiO_4_ started to collapse at higher temperatures in the ZnO-SiO_2_ composite. The absorption edges became smaller and, thus, decreased the optical band gap [32,33,34]. On the other hand, the obtained optical band gap value is in agreement with a previous study where pure SiO_2_ was used [35], which resulted in the band gap being indirect. Therefore, it can be concluded that palm waste has high potential to be an alternative that can replace SiO_2_ to produce the composite.

## 3. Materials and Methods

An empty fruit bunch was first cleaned using distilled water and then dried up in an oven at 70 °C for 24 h. After that, the fruit bunch was crushed and burned in a furnace at 600, 700 and 800 °C for 2 h. The palm ash obtained was cooled down inside the furnace and collected for a preliminary quantitative analysis. Quantitative analysis of the sample chemical compositions was carried out using an X-ray fluorescence (XRF) XRF-EDX-720/7000 machine (Shimadzu, Kyoto, Japan).

Next, the palm ash sample with the highest percentage of silica (800 °C) was selected for further experiments. The sample underwent acid treatment, where 20 mL of 5 N (normality) sulfuric acid was added to the sample. The mixture was stirred using a hot plate magnetic stirrer at 50 °C for 30 min and then filtered using Whatmann No. 41 ashless filter paper. Finally, the residue was dried in an oven at 60 °C for 1 h to obtain the sample in powder form. XRF analysis was conducted once again to confirm the chemical composition of the sample after the acid leaching process. Phase identification of the samples was conducted by using an X-ray diffraction (XRD) Phillips X’Pert High Pro PANanalytical Diffractometer (Malvern Pananalytical, Almelo, (The Netherlands) and Malvern (UK)), with a data range of 2θ from 20° to 80°. To analyze the morphological structure of the samples, field emission scanning electron microscopy (FESEM) (Nova NanoSEM 30 (FEI, Hillsboro, OR, USA)) was used.

To prepare the ZnO-SiO_2_ composite, a conventional solid-state method was used. Acid-treated palm waste silica and ZnO nanopowders (US Research Nanomaterials, Inc., United State of America, 99%+, 10–30 nm ZnO) were mixed in the ratio 1:1 via milling process using a ball milling jar for 24 h. After the milling process, the mixture was then sintered in an alumina crucible at 600 to 1400 °C at a heating rate of 10 °C/min for 2 h in an electrically heated furnace. One sample remained at room temperature, 27 °C. Structural characterization of the composite sintered at various temperatures was analyzed using XRD and FESEM. For optical characterization, UV–Vis absorption analysis was carried out using a UV-3600 Shimadzu spectrophotometer (Shimadzu, Kyoto, Japan). The absorption spectrum obtained was used to calculate the optical band gap for the materials.

## 4. Conclusions

This study investigated oil palm empty fruit bunch (OPEFB) as an alternative resource for silica. An OPEFB that was burned at 800 °C was then leached to remove impurities and increase the percentage of silica composition in the sample. The XRF result shows that the percentage of silica increased from 9.5% to 45.6% after leaching. For ZnO-SiO_2_, the XRD result showed that intensity was the highest at 1000 °C as compared to other sintering temperatures. Most of the diffraction peaks were indexed to the hexagonal structure of ZnO. For the surface morphology of ZnO-SiO_2_, the result showed that the grain boundaries started to increase as the temperature raised, resulting from the crystal growth. For UV–Vis analysis, the results showed that a sharp absorption edge was observed at a wavelength of about 360 nm at room temperature. When the prepared sample was sintered at temperatures from 600 to 1400 °C, a broad absorption band appeared in the range of 270 to 310 nm. Both direct and indirect energy band gaps of the composite increased from room temperature to 800 °C and started to decrease from 1000 to 1400 °C because the formation of Zn_2_SiO_4_ started to collapse at higher temperatures for the ZnO-SiO_2_ composite, with an indirect energy band gap close to the composite formed by pure silica. In short, OPEFBs have high potential as a silica source to be used in optical materials.

## Figures and Tables

**Table 1:** Chemical composition of palm ash derived from oil palm empty fruit bunch (OPEFB).

Compounds	Percentage of Compositions		
	600 °C	700 °C	800 °C
CaO	40.0	52.2	39.2
K_2_O	40.2	29.8	35.2
SiO_2_	5.6	6.9	9.5
P_2_O_5_	2.9	4.2	4.2
Fe_2_O_3_	2.4	1.5	2.6
Cl	5.9	3.0	2.5
SO_3_	1.7	1.3	2.1

**Table 2:** Chemical composition of palm ash (before and after leaching).

Compounds	Percentage of Compositions	
	Before	After
CaO	39.2	17.8
K_2_O	35.2	16.4
SiO_2_	9.5	45.6
P_2_O_5_	4.2	0.0
Fe_2_O_3_	2.6	3.3
Cl	2.5	0.0
SO_3_	2.1	3.5

## Data Availability

The data presented in this study are available within the article.
